# A Case Report of a Transected Carotid Artery Caused by a Stab Wound to the Neck

**DOI:** 10.21980/J8BP8M

**Published:** 2021-01-15

**Authors:** Jennifer Roh, Kylie Prentice

**Affiliations:** * University of California, Irvine, Department of Emergency Medicine, Orange, CA

## Abstract

Penetrating wounds to the neck occur in 5–10% of trauma cases and are associated with very high mortality due to proximity to and involvement of vital structures. Emergent airway management and rapid diagnosis in these injuries are essential for life-saving treatment. This case study presents the case of a stab wound to the neck in an adult patient which elicited major concern for cardiovascular, neurological, and airway compromise. In such a high-risk trauma airway, this case shows the importance of keeping a wide differential and the value of expeditious management to prevent anoxic brain injury and stroke. The rapid diagnosis of a left common carotid artery transection and subsequent surgical repair were essential in this patient’s full neurological recovery.

**Topics:**

Trauma, stab wound, neck hematoma, deviated trachea, carotid artery injury, carotid artery transection.

**Figure:**
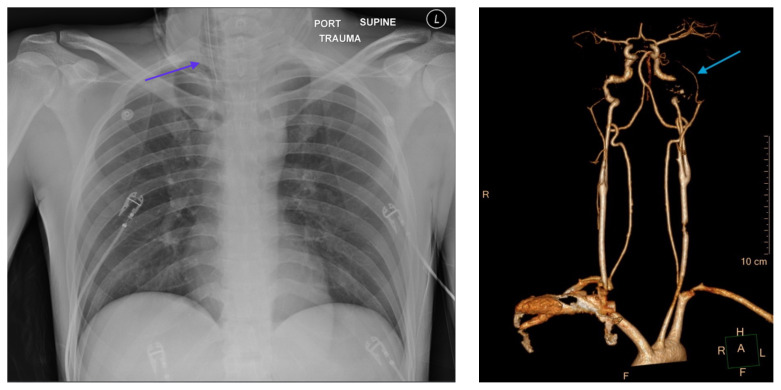

**Figure:**
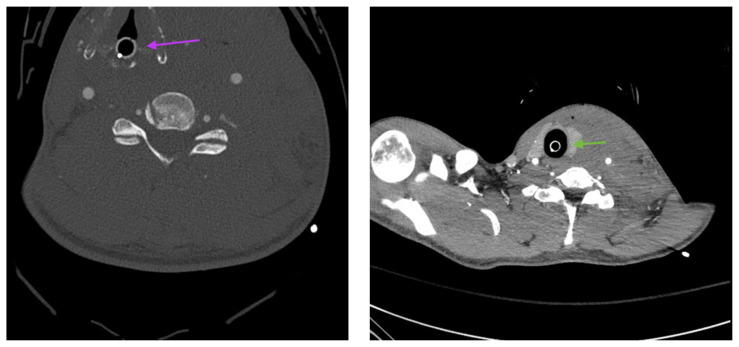

## Brief introduction

Penetrating injuries to the neck represent 5–10% of all trauma cases and have a mortality of 2–10% because of the vital structures in the neck.^1^,^2^ Vascular injuries correlate with poorer outcomes^2^ and accurate diagnosis is important for specific management of these high-risk injuries. Computed tomography angiography (CTA) is the first-line diagnostic tool for vascular injuries in hemodynamically stable patients.^3^ In this report, we present a transected carotid artery secondary to a stab wound and its subsequent management.

## Presenting concerns and clinical findings

A 28-year-old male presents to the emergency department as a critical trauma activation with a single penetrating wound to his neck. The verbal report given by paramedics prior to arrival to the Emergency Department (ED) was that the patient had a low Glasgow Coma Scale (GCS) of 3 and an anterior stab wound to the trachea. When the patient arrived to the ED, he had moderate altered mental status and could not vocalize, but was able to open eyes on command. His GCS upon arrival was 8, and he had difficulty breathing. There was severe swelling covering the entirety of his anterior neck as well as a small non-bleeding horizontal laceration in the center of the anterior neck. The swelling was so significant that no anterior anatomical structures were visible from the angle of the mandible to the cricoid cartilage (Zone II). The patient had equal breath sounds bilaterally and pulses intact in all extremities. He was not bleeding actively from the neck wound. Initial vital signs were blood pressure 113/70 mm Hg, pulse 112, respiratory rate 18 breaths per minute and oxygen saturation of 100% on a non-rebreather mask. The emergency physicians had prepared for rapid sequence intubation with multiple back-up methods including equipment to perform a surgical airway if needed. The patient was successfully intubated with a Glidescope on the first attempt.

## Significant findings

The post intubation chest x-ray (CXR) showed severe rightward displacement of the trachea (purple arrow). The computed tomography angiogram (CTA) showed transection of the left common carotid artery (LCCA), extensive neck hematoma without extravasation and severe tracheal deviation to the right (blue arrow). The intravenous (IV) contrasted chest computed tomography (CT) image showed a lateral contrast projection from the aortic arch at the level of the isthmus (green and pink arrows). There were no other significant injuries reported on the CT scans of the chest, abdomen and pelvis.

## Patient course

After emergent CT and CTA imaging, the patient was taken immediately to the operating room. The patient underwent a neck exploration given high concern for carotid artery injury, and well as possible damage to other critical structures. The transected LCCA was confirmed and repaired and there was no damage to other neck structures. After surgery he was transferred to the Surgical Intensive Care Unit (SICU). Further workup showed positive serum ethanol and methamphetamine in the urine, blood loss anemia and lactic acidosis which later resolved. Approximately eight hours later the patient started to breathe spontaneously and was found to be neurologically and neurovascularly intact. He was extubated soon after, but was kept NPO due to dysphagia. Two days post-operatively he downgraded to the ward and started a diet with thin liquids. His voice and mental status returned to normal, and after two more days he was discharged home in good condition.

## Discussion

In the United States, penetrating neck injuries (PNI), defined by the breach of the platysma muscle, can involve any of the major structures in Zone I, II, and III. Most PNI are caused by stab wounds or gunshot wounds.^4^,^5^ Less common causes of PNI include vehicle accidents and injury from high-velocity objects.^5^ Arterial vascular injuries make up 25% of PNI, and the carotid artery is involved in 80% of injuries^4^ which can lead to stroke like symptoms, airway compression, and critical blood loss. Aerodigestive injuries are seen in 23–30% of PNI and are also associated with high mortality.^6^ The overall mortality for PNI is between 2–10%. Hard signs of vascular injury to the neck include shock, pulsatile bleeding, expanding hematoma, audible bruit or palpable thrill, airway compromise, wound bubbling, subcutaneous emphysema, stridor, hoarseness, difficulty tolerating secretions, and neurologic deficits. 9 Traditionally, patients with any of these signs would be taken directly to the OR (the zone based algorithm)^9^; however, several studies by trauma surgeons have questioned the traditional approach and argue that injuries can extend beyond the wound and that imaging can help with surgical management.^9^,^10^ CTA has an overall sensitivity of 97% and overall specificity of 99% and is considered the gold standard in diagnosing vascular injuries in neck trauma.^3^ In this case, the surgeons opted to take the patient for immediate CTA and CT imaging on the way to the OR.

This case is an important example of a high-risk trauma airway. It illustrates the importance of being prepared to immediately address airway emergencies in penetrating trauma to the neck. In this case, this patient's underlying pathology was a transected carotid artery. It was suspected that there was an arterial injury causing a large hematoma and likely tracheal deviation; however, the entire anterior neck was swollen and structures were not visible. It was unclear if the trachea was also transected or if there was also injury to the larynx or also the esophagus. The emergency physicians were prepared for a variety of airway complications; however, in this case, a cricothyroidotomy would be less ideal given the high likelihood for arterial vascular injury. Alternative methods to direct endotracheal intubation are bougie-assisted intubation, fiberoptic intubation, or laryngeal mask airway (LMA), and Combitube (esophageal-tracheal twin-lumen airway device) insertion as a bridge to surgical tracheostomy repair of the airway.^8^ If the trachea was transected without an obscuring hematoma, an ET tube could be inserted directly through the tracheal defect as an alternative pathway to oral intubation^10^. Emergent airway management as well as accurate diagnosis of the transected LCCA led to the timely repair of the LCCA by trauma surgeons. Expeditious management helped avoid anoxic brain injury secondary to airway compromise and also avoided permanent stroke from poor brain perfusion secondary to the transected LCAA. The patient was able to have a full recovery without any neurologic deficits.

## Supplementary Information
















