# Supplementary figures and images for: A Case Report of a Transected Carotid Artery Caused by a Stab Wound to the Neck
Source: J Educ Teach Emerg Med. 2021 Jan 15;6(1):V15–8. doi: 10.21980/J8BP8M (PMC10332755; doi:10.21980/J8BP8M)

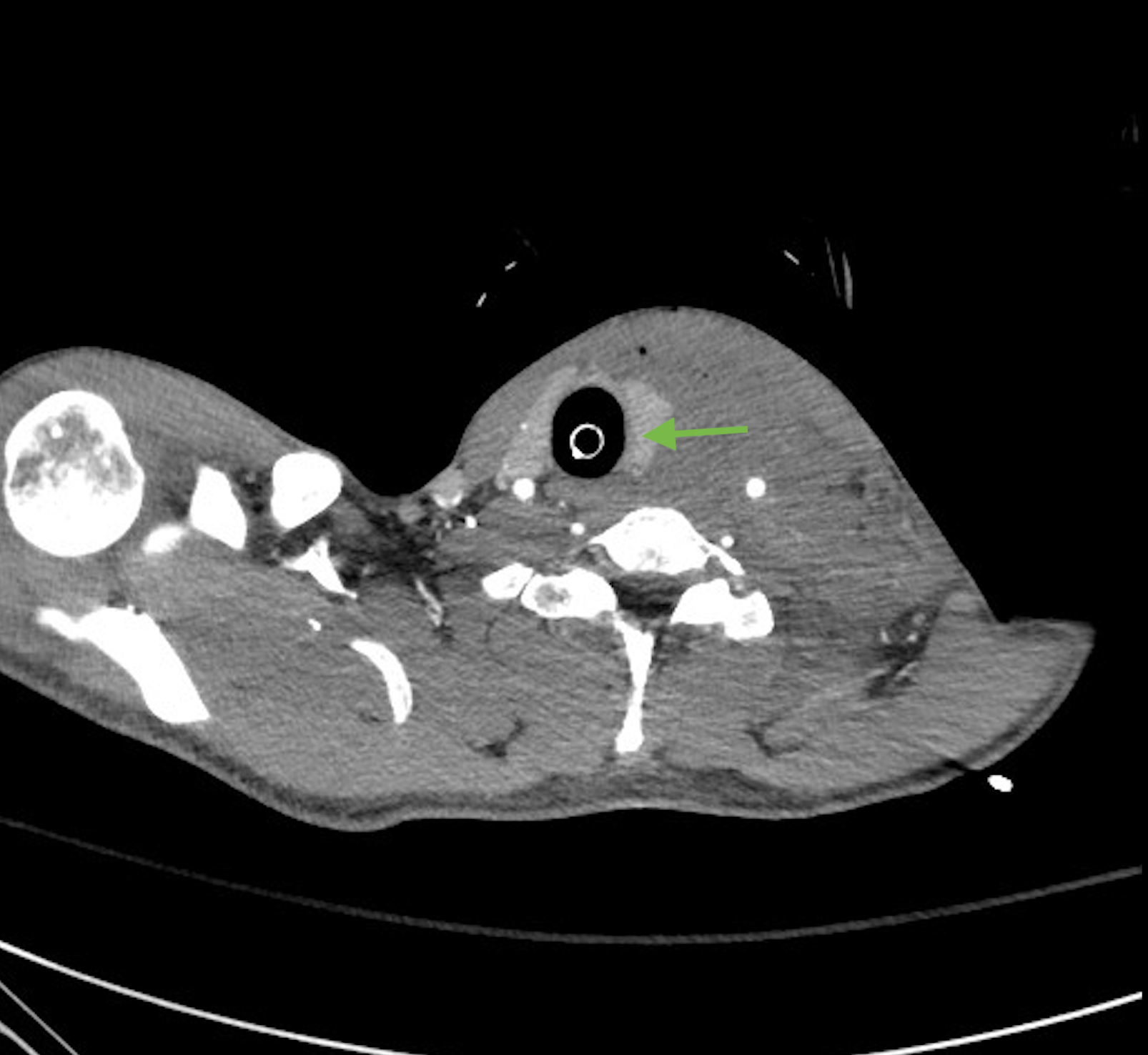

Supplement: Supplementary file 1 [file jetem-6-1-v15-supp1.jpeg]

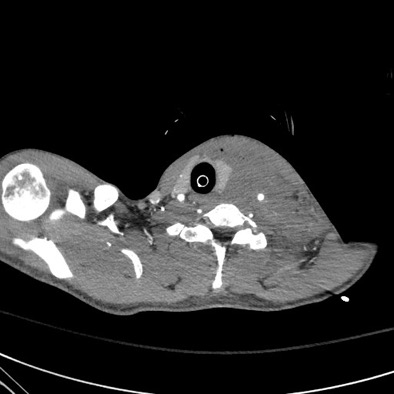

Supplement: Supplementary file 2 [file jetem-6-1-v15-supp2.jpg]

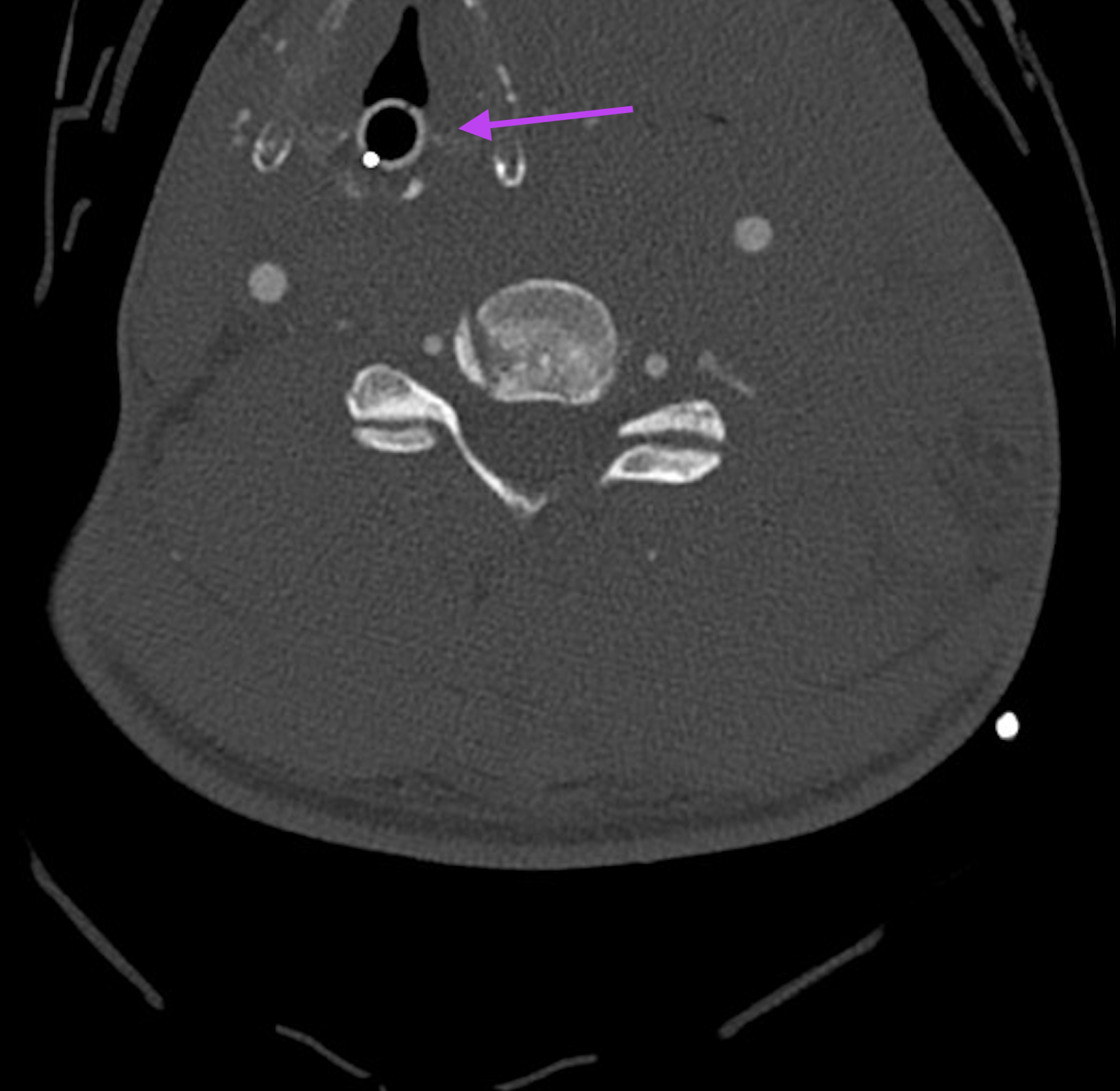

Supplement: Supplementary file 3 [file jetem-6-1-v15-supp3.jpeg]

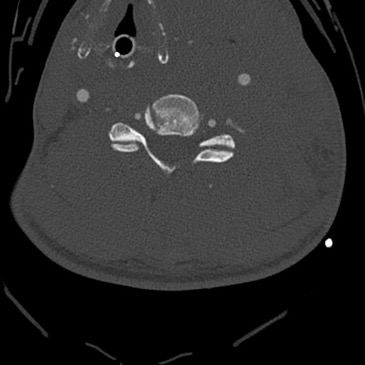

Supplement: Supplementary file 4 [file jetem-6-1-v15-supp4.jpg]

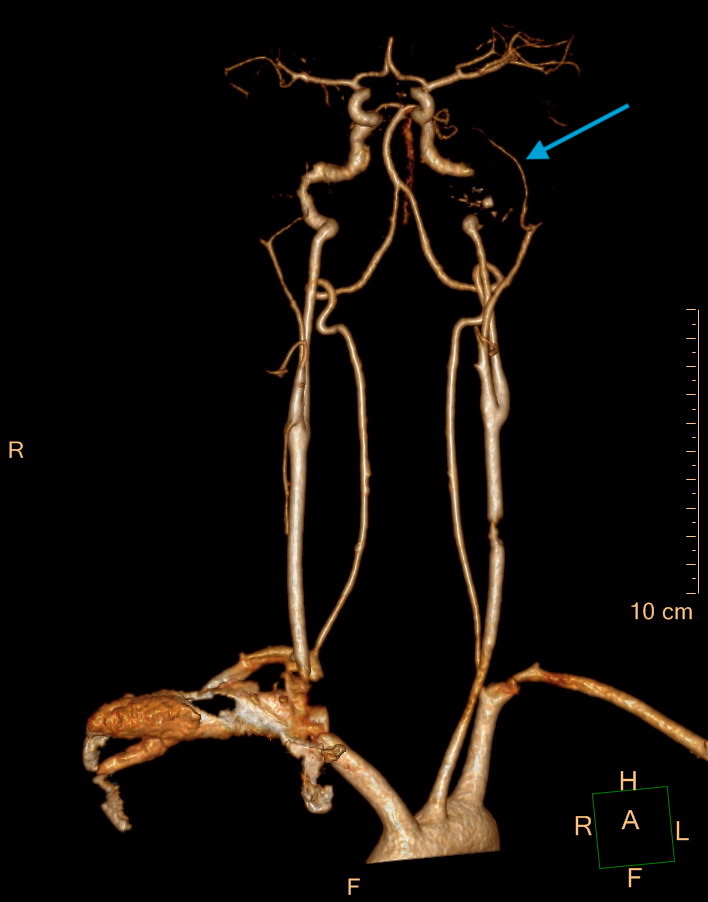

Supplement: Supplementary file 5 [file jetem-6-1-v15-supp5.jpeg]

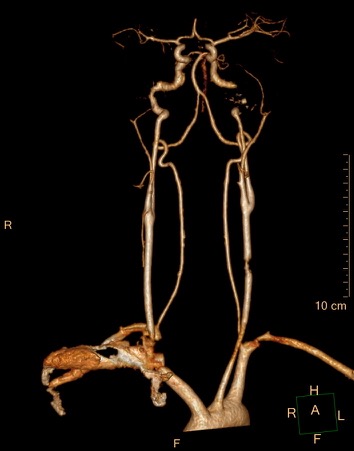

Supplement: Supplementary file 6 [file jetem-6-1-v15-supp6.jpg]

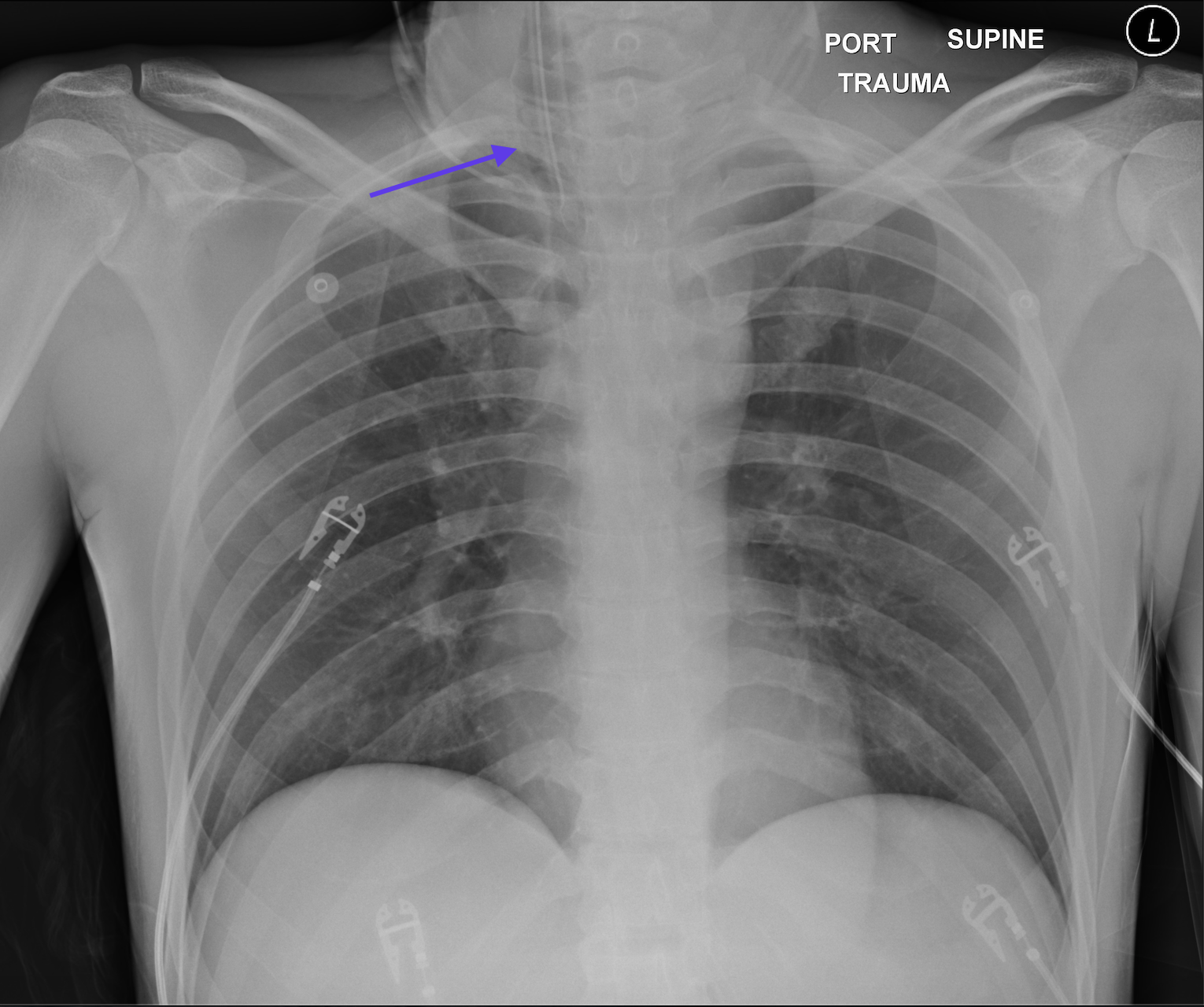

Supplement: Supplementary file 7 [file jetem-6-1-v15-supp7.jpeg]

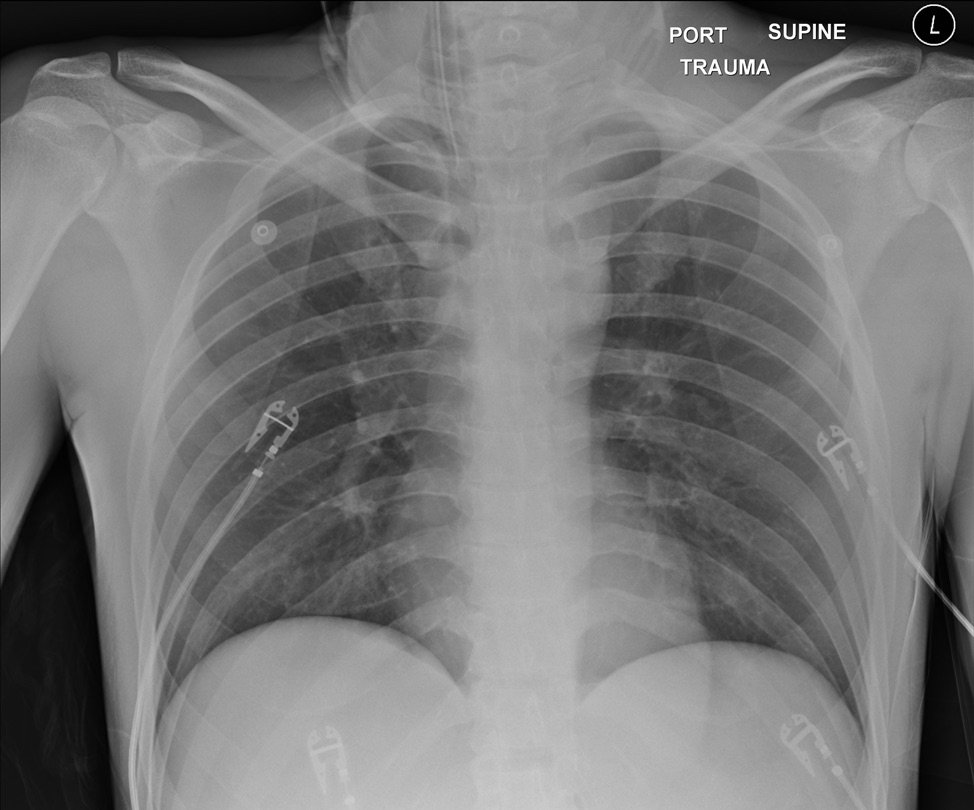

Supplement: Supplementary file 8 [file jetem-6-1-v15-supp8.jpg]
